# Precise Dissolution Control and Bioavailability Evaluation for Insoluble Drug Berberine via a Polymeric Particle Prepared Using Supercritical CO_2_

**DOI:** 10.3390/polym10111198

**Published:** 2018-10-26

**Authors:** Jingfu Jia, Kerong Zhang, Xue Zhou, Dan Zhou, Fahuan Ge

**Affiliations:** 1 School of Pharmaceutical Sciences, Sun Yat-sen University, Guangzhou 510006, China; jiajingfu@mail.sysu.edu.cn (J.J.); 15388779694@163.com (K.Z.); zhouxue9@mail.sysu.edu.cn (X.Z.); 2 Nansha Research Institute of Sun Yat-Sen University, Guangzhou 511458, China; zhoudandanny@163.com

**Keywords:** berberine, polymeric particle, supercritical CO_2_, dissolution, bioavailability

## Abstract

It is still controversial whether poor aqueous solubility is the most primary reason for the low oral bioavailability of insoluble drugs. Therefore, in this study, berberine-loaded solid polymeric particles (BPs) of varied dissolution profiles with β-cyclodextrin (β-CD) as carrier were fabricated using solution-enhanced dispersion by supercritical fluids (SEDS), and the relationship between dissolution and berberine (BBR) bioavailability was evaluated. Dissolution property was controlled via particle morphology manipulation, which was achieved by adjusting several key operating parameters during the SEDS process. Characterization on BP using infrared spectroscopy, differential scanning calorimetry, and X-ray diffraction indicated that BBR was dispersed in amorphous form, while nuclear magnetic resonance spectroscopy showed that methoxy groups of BBR were included into the cavities of β-CD. In vivo pharmacokinetic studies showed that oral bioavailability increased by about 54% and 86% when the dissolution rate of BBR was increased by 51% and 83%, respectively. The entry speed of BBR into the bloodstream was also advanced with the degree of dissolution enhancement. It seemed that dissolution enhancement gave positive effect to the oral bioavailability of berberine, but this might not be the crucial point. Meanwhile, supercritical CO_2_ technology is a promising method for pharmaceutical research due to its advantages in regulating drug-dosage properties.

## 1. Introduction

Berberine (BBR), a representative quaternary protoberberine isoquinoline alkaloid generally existing in the form of hydrochloride, is one of the most important natural medicines that can be extracted from plants like *Coptis* sp. or *Berberis* sp. [1]. BBR was early considered in clinics as an effective and safety agent widely used for the treatment of diarrhea and gastroenteritis [2], due to its pharmacological antibacterial [3], anti-inflammatory [4,5] and antiparasitic [6] properties. In recent years, it has reported that BBR also possesses antihypertension [7], antidiabetic [8], and antitumor [9,10] qualities, and helps in reducing cholesterol and lipid accumulation in both the plasma and liver [11], further confirmed in clinic [12]. However, the very low oral bioavailability of BBR (below 1%) seriously limits the clinical utilization of these pharmacological qualities, and poor adsorption in the intestine is generally considered the main affecting factor, except for the first-pass effect in the liver [13]. Furthermore, the poor absorption of BBR may be attributed to the drug’s physicochemical properties, including self-aggregation, poor permeability, low dissolution, and physiological factors like P-glycoprotein-mediated efflux [13].

In order to enhance the bioavailability of BBR, several strategies from different perspectives have been reported, such as using anionic surfactants (e.g., sodium caprate and sodium deoxycholate) or cationic polysaccharide (e.g., chitosan) as permeability enhancers [14,15,16], adding cosolvents (e.g., polyethylene glycol [17]) or surfactants (e.g., d-α-tocopheryl polyethylene glycol 1000 [18]) as P-gp inhibitors to avoid the efflux of P-gp, preparing inclusion complexes using β-cyclodextrin (β-CD) and its derivatives [19,20,21] to enhance the dissolution properties of BBR, and fabricating lipid micro/nanodrug delivery systems that possess multiple functions [22,23,24,25]. In these studies, the oral bioavailability increase greatly varied, from 1.2- to 41.1-fold, even using a similar dosage form, which may be caused by the complexity of the drug-absorption mechanism. In other words, the influence extent of each factor is not yet clear, making it unclear how to design a dosage to further improve the oral bioavailability of BBR.

Therefore, in this study, we attempted to prepare berberine micro- and nanoparticles (BPs) with varied dissolution properties to explore the relationship between dissolution enhancement and the oral bioavailability of BBR. β-CD was used as a drug carrier, and traditional inclusion complexes of BBR (ICB) were also prepared for comparison. A supercritical antisolvent process (SAS), which has attracted widespread attention and research in the pharmacological field over the years, has been proven to be a convenient, product-controlled, and environmentally friendly method for preparing micro- or nanoparticles [26]. Through this green approach, nature-controllable and organic solvent-free nanoparticles with high entrapment efficiency can be obtained [27]. Thus, solution-enhanced dispersion by supercritical fluids (SEDS), a cutting-edge SAS method, was chosen to prepare BPs and control their dissolution properties through morphology manipulation. Several key parameters were investigated, and particle structure was characterized. On this basis, the relationship between the dissolution properties of BBR and oral bioavailability was studied through in vivo pharmacokinetic studies in rats.

## 2. Materials and Methods

### 2.1. Materials and Animals

Berberine hydrochloride (≥95.0%) and β-cyclodextrin (99.0%) were purchased from Aladdin (Shanghai, China). Carbon dioxide (99.9%) was obtained from Guangzhou Gas Factory Co., Ltd. (Guangdong, China). Acetonitrile of chromatographic purity was obtained from Merck Ltd. (Darmstadt, Germany). Other organic solvents, like dimethyl sulfoxide (DMSO) and dichloromethane (DCM), used in this research were analytically pure.

Male pathogen-free SD rats, 250 ± 10 g, were bought from the Experimental Animal Center in Guangzhou University of Chinese Medicine (Guangzhou, China). The standard feeding conditions for rats were a temperature of 25.0 ± 1.0 °C, humidity of 60% ± 10%, and 12 h light/dark cycles. Standard diet and water were freely available. The animals were used following the guidance of the Ethical Committee for Animal Experiments of Guangzhou University of Chinese Medicine (SCXK (Yue) 2013-0034).

### 2.2. Preparation of BPs and ICB

The experimental apparatus used for preparing BPs via SEDS was shown in our early work [28], and the procedure can be briefly described as follows: Firstly, pure CO_2_ was conveyed at an invariable flow rate (5 L/h) from a gas cylinder (initial pressure of 55 bar) to a precipitation chamber via a condenser (4 °C) and a preheater of preset temperature. Then, CO_2_ kept entering the precipitation chamber through the inside tube of a coaxial nozzle (RZN-120 and RZN-160, Nantong Ruizhi Supercritical Development of Technology Co., Nantong, China) to reach the required pressure. Meanwhile, the precipitation chamber maintained the same temperature via a temperature-controlled heating mantle. Then, keeping pressure stable, a solution of BBR and β-CD in mixed solvents of DMSO and DCM (1:1, *v*/*v*) was injected into the chamber at 1 mL/min until the particle-precipitation process was completed. After that, CO_2_ kept flowing at least two times the solution-feeding duration to entirely remove residual solvent. In the end, the system was slowly depressurized, and the particle powder was collected in the chamber, where a stainless mesh was located at the bottom to prevent the escape of particles. The organic solvent was recovered with a recycle tank connected at the end.

ICB were also prepared for comparison using a saturated-aqueous solution method. Concisely, an accurately weighed amount of β-CD was dissolved in water to obtain a saturated, colorless, and transparent solution (72.9 mg/mL). Then, BBR dispersed in ethanol (119 mg/mL) was added slowly to the solution and stirred (400 rpm) at 60 °C for 5 h. Finally, the orange aqueous mixture was cooled at room temperature and stored at 4 °C for another 8 h, and the resultant brownish precipitation was separated and dried at 60 °C to obtain the brown ICB powder.

### 2.3. HPLC Analysis of BBR Content

The BBR contents in BPs were analyzed by a high-pressure liquid chromatography system (HPLC, UltiMate 3000, Thermo Fisher Scientific Ltd., Waltham, MA, USA). According to Chinese pharmacopoeia (2015), a Bluesil C18 chromatography column was used, and an acetonitrile/potassium dihydrogen phosphate aqueous solution (0.01 mol/L, pH = 2.80) (35/65) was employed as the mobile phase. BBR was detected by UV at 245 nm, the volume was maintained at 40 °C, and the flow rate was 1 mL/min.

### 2.4. Characterization Methods

The surface morphologies of BPs, as well as the raw BBR and ICB, were observed using a field emission scanning electron microscope (SEM, JSM-6330F, JEOL Ltd., Tokyo, Japan) with platinum coating for 120 s by a sputter coater (1.2 kW, E-1045, Hitachi, Tokyo, Japan).

The chemical structure was characterized by a Fourier transform infrared spectrometer (FTIR, Equinox 55, Bruker (Beijing) Technology Co., Ltd., Beijing, China) between wave numbers of 4000 and 400 cm^−1^ using the KBr disc technique. Additionally, the X-ray powder diffraction patterns (XRD) were plotted with an X-ray diffractometer (Empyrean, PANalytical B.V., Almelo, The Netherlands) within the 2 *θ* angle range from 3.0° to 50.0°. Thermal behaviors were also analyzed with a differential scanning calorimeter (DSC, DSC-204, Netzsch, Selb, Germany) under a nitrogen atmosphere from 30 to 250 °C at a rate of 10 °C/min.

Nuclear magnetic resonance (NMR) spectra (1D and 2D) of the raw BBR, β-CD, ICB and BP were recorded on a Bruker Avance-600 MHz spectrometer (Bruker (Beijing) Technology Co., Ltd., Beijing, China) equipped with a TCI CryoProbe in D_2_O at 25 °C using tetra methyl saline as internal standard. Two-dimensional NMR studies, nuclear overhauser effect spectroscopy (NOESY), were performed in phase-sensitive mode. The 2D spectra were acquired with free induction decays (FID) containing 32 scans with relaxation delays of 1.5 s. The NOESY experiments were performed with mixing time of 0.2–0.3 s. The two-dimensional data were processed with Gaussian apodization in both the dimensions.

### 2.5. Dissolution Study

According to Chinese pharmacopoeia (Part IV, 2015), dissolution measurements of BPs, raw BBR, ICB and physical mixture (BBR/β-CD, *n*/*n*, 1:1) were carried out for 80 min in 50 mL phosphate-buffered saline (PBS, pH 6.8) and HCl solution (pH 1.2), separately, at 37 °C with constant stirring at 25 rpm using a dissolution tester (RCZ-8 M, TDTF, Tianjin, China). After placing each sample (amounting to 400 mg of BBR) into the dissolution vessel, 0.7 mL samples were collected at proper time intervals and filtered through a 0.45 μm microfiltration membrane. PBS of the same volume was replenished after each sample withdraw. The samples were then characterized using under voltage (UV).

### 2.6. Pharmacokinetic Studies in Rats and Data Analysis

Eighteen rats were randomly divided into 3 groups with 6 rats in each, fasting from solids but free for water 12 h before the experiment. The 3 groups were administered berberine, suspensions of micro-BP (BP_m_) and nano-BP (BP_n_) (dispersed in saline solution) at a berberine dose of 150 mg/kg, respectively. The blood samples (0.6 mL) were withdrawn from the orbital cavity and collected in an anticoagulant centrifugal tube at different time intervals. Plasma was separated by centrifuging the samples at 5000 rpm for 10 min at 4 °C and immediately stored at −20 °C for analysis. Acetonitrile (300 μL) was added to rat plasmas (100 μL), and vortexed for 90 s, then centrifuged (12,000 rpm for 10 min). Two-hundred microliters of supernatant was injected into liquid chromatography-mass spectrometry (LC-MS, TSQ QUANTUM ACCESS MAX, Thermo Fisher Scientific, Waltham, MA, USA) for the determination of berberine in plasma.

Peak concentration (*C*_max_) and time of peak concentration (*T*_max_) were obtained directly from the individual plasma concentration–time profiles. The area under the concentration–time curve (*AUC*) from time zero to test time (*AUC*_0→t_) was calculated using the trapezoidal method. The *AUC* from zero to infinity (*AUC*_0→__∞_) was calculated by trapezoidal rule for the observed values and subsequent extrapolation to infinity. The relative bioavailability was *F*_rel_ = (*AUC*_0→__∞_ of BP/*AUC*_0→__∞_ of BBR) × 100%. 

## 3. Results and Discussion

### 3.1. Particle Morphology Manipulation

**Figure 1:**
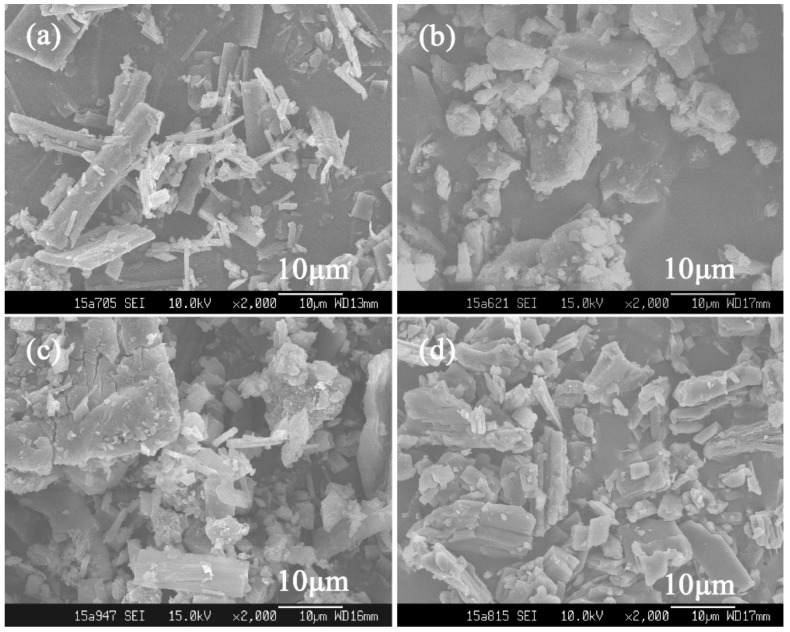
Scanning electron microscopy (SEM) images of (**a**) raw berberine (BBR); (**b**) β-cyclodextrin (β-CD); (**c**) a physical mixture (1:1, *n*/*n*) of BBR and β-CD; and (**d**) inclusion complexes of BBR (ICB).

The SEM images of raw materials in Figure 1 show that the particle shapes of raw BBR (Figure 1a) are prismatic, while those of raw β-CD (Figure 1b) are massive. Both shapes can be observed in the physical mixture of BBR and β-CD (Figure 1c), as well as in ICB (Figure 1d). The similar appearance of ICB to the physical mixture indicates the form of ICB hardly changed the morphologies of the raw materials. Furthermore, BBR was only partially embedded in β-CD during the ICB preparation process using the saturated–aqueous solution method.

**Figure 2:**
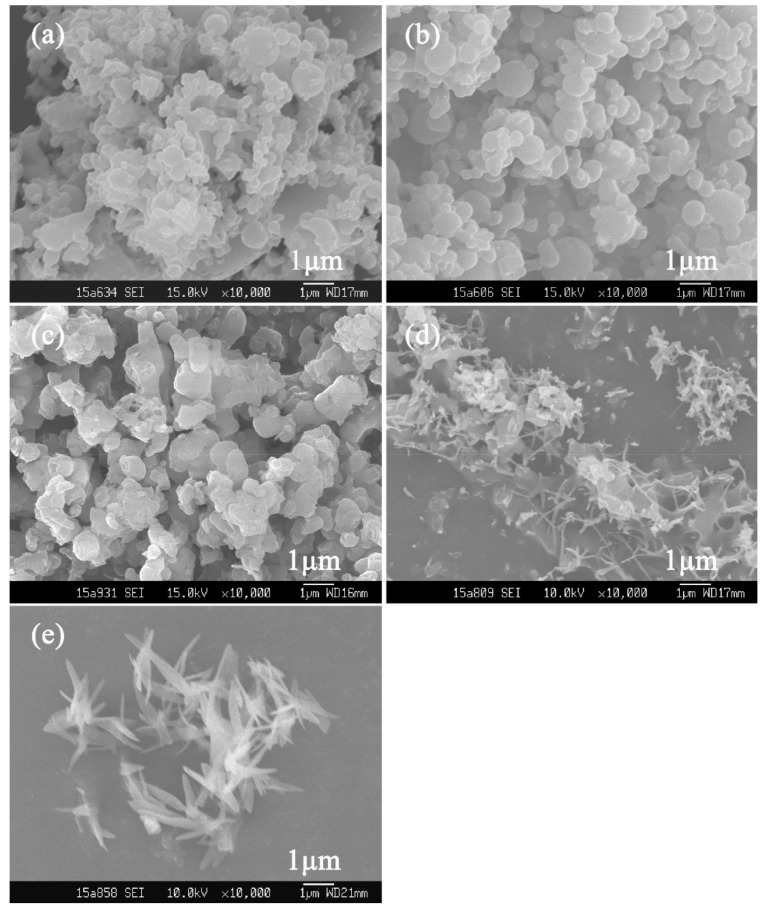
SEM images of a berberine-loaded particle (BP) prepared under a different mole ratio (*λ*) of BBR to β-CD via solution-enhanced dispersion by supercritical fluids (SEDS): (**a**) 1:2, Sample 1; (**b**) 1:1, Sample 2; (**c**) 3:1, Sample 3; (**d**) 5:1, Sample 4; and (**e**) BBR only, Sample 5.

**Figure 3:**
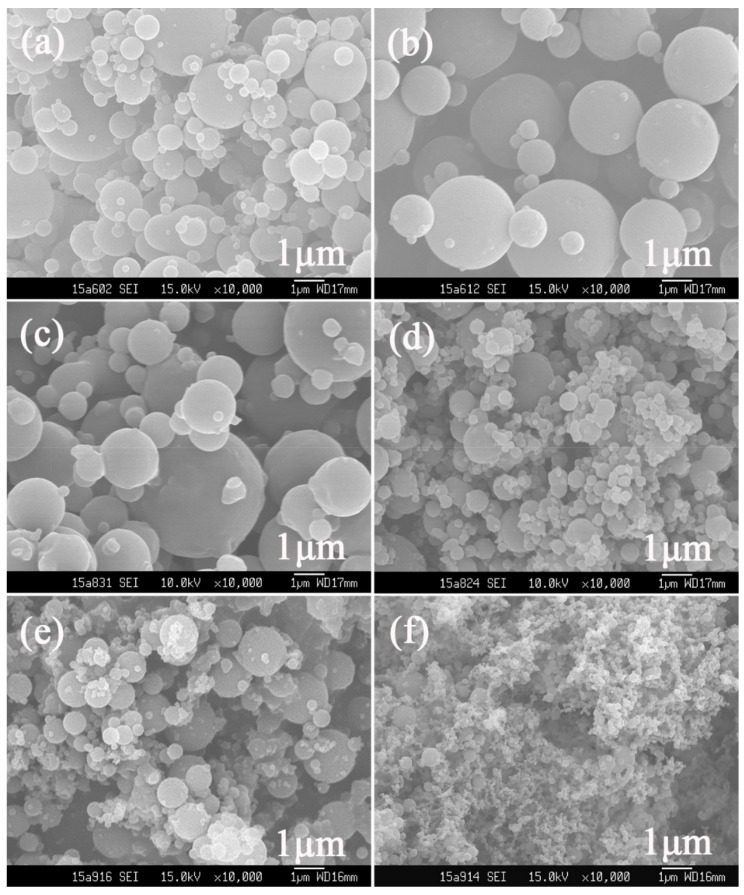
SEM images of a BP prepared under different conditions (temperature, concentration, pressure): (**a**) 50 °C, 120 bar, 15 mg/mL, Sample 6; (**b**) 60 °C, 120 bar, 15 mg/mL, Sample 7; (**c**) 40 °C, 90 bar, 15 mg/mL, Sample 8; (**d**) 40 °C, 150 bar, 15 mg/mL, Sample 9; (**e**) 40 °C, 120 bar, 9 mg/mL, Sample 10; (**f**) 40 °C, 120 bar, 3 mg/mL, Sample 11.

In contrast, the appearance of BPs obtained via SEDS shown in Figure 2 and Figure 3 turned out to not only be quite different from the raw materials, but also to have diversity among themselves. The morphology of particles prepared via SEDS could be manipulated by adjusting the operating parameters, which has been proven by numerous studies [29,30,31]. Therefore, parameters including the mol ratio of BBR to β-CD (defined as *λ* hereinafter), temperature, pressure, and solution concentration of BBR were investigated to obtain particles of varied shapes and sizes, which is summarized in Table 1.

#### 3.1.1. Effect of *λ*

Since the formula of drug and carrier is a cardinal parameter impacting on the particle morphology for preparing hybrid micro- or nanoparticles via the SEDS process [32], *λ* was primarily investigated. Figure 2 shows the SEM images of particles obtained at an *λ* of 1:2, 1:1, 3:1, and 5:1 with BBR concentration fixed at 15 mg/mL, as well as BBR processed without β-CD (BBR crystal). The pressure and temperature were fixed at 12 MPa and 40 °C, respectively.

It can be seen that, with the decrease of the amount of β-CD, particle morphology shifted from spherical nanoparticles (i.e., BP_n_, Figure 2a,b) to irregular (Figure 2c), and a mixture of irregular and needle particles (BP_mix_, Figure 2d) in the end. Referring to the needlelike shape of the BBR crystal (Figure 2e), it is rational to conclude that, in BP_mix_ produced at an *λ* of 5:1 (Figure 2d), most BBR precipitated in a crystal morphology by itself, rather than mingling with β-CD in a hybrid form. Interestingly, needle particle size was much smaller than that of BBR processed alone. However, at an *λ* of 1:2 or 1:1, uniform hybrids of BBR and β-CD appearing as spherical nanoparticles were produced. This was because, during the process of BP formation via SEDS, where a nucleation and growth mechanism dominated, the crystal growth of BBR could be inhibited by β-CD, and inhibitory intensity increased with the amount of used β-CD (e.g., transitional morphology could be observed at an *λ* of 3:1 in Figure 2c). This phenomenon of crystal-growth inhibition by carrier materials towards crystal drugs in a supercritical antisolvent process has also been reported elsewhere [32,33]. Considering maximizing the drug load, an *λ* of 1:1 was selected for further investigation. 

#### 3.1.2. Effect of Temperature, Pressure, and Concentration

At an *λ* of 1:1, berberine-loaded particles prepared under adjusted temperature, pressure, and concentration are shown in Figure 3, where spherical particles were obtained in all operating conditions, but with varied particle sizes. 

The SEM images of particles prepared at different temperatures are shown in Figure 2b (40 °C), Figure 3a (50 °C), and Figure 3b (60 °C), respectively. Particle size increased with temperature elevation from nanosized to the micro scale. A similar particle-size tendency occurred when pressure decreased, that is, BP_n_ were obtained at a pressure of 12 (Figure 2b) and 15 (Figure 3d) MPa, while BP_m_ were collected at 9 MPa (Figure 3d). This particle-size variation is linked with the mass transfer between CO_2_ and liquid solvents in the SEDS process, of which the duration can hardly be ignored, especially when high-viscosity solvents (e.g., DMSO) are used [34,35]. Due to this mass transfer, a local supersaturation gradient exists, leading to a wide-size span for particles formed based on the nucleation mechanism [36]. Generally, the slower the mass transfer speed is, the wider the size span is and the bigger the obtained particles are. Furthermore, mass transfer speed increases with the solvability of supercritical CO_2_, which has a positive correlation with its density. During the process, the density of supercritical CO_2_ decreased with the temperature increase or pressure reduction, resulting in a slower mass transfer between CO_2_ and DCM/DMSO. Thus, bigger and nonuniform particles were collected at a higher temperature (i.e., 50 °C and 60 °C) or lower pressure (i.e., 9 MPa).

Products using lower BBR concentrations are shown in Figure 3e (9 mg/mL) and Figure 3f (3 mg/mL). It is obvious that particle size reduced with the concentration decrease, and uniform BP_n_ of about 200 nm were generated at a BBR concentration of 3 mg/mL. This size change can be attributed to the accelerated mass transfer caused by the dropped solution viscosity [36], born of a less-used amount of β-CD. 

### 3.2. Composition Analysis

The BBR contents in the prepared particles were analyzed by HPLC and summarized in Table 1. The actual mol ratios of BBR to β-CD (*λ′*) in products appeared biased to those values of *λ*, attributed to the different recoveries of BBR and β-CD in SEDS. However, *λ′* was close to *λ* when the latter was not bigger than 1:1, while deviations became significant above (and including) 3:1, indicating BBR loss. This may be because partial BBR was not embedded in β-CD. and the free BBR was easier to be removed with supercritical CO_2_ than β-CD, making *λ′* less than *λ*. 

For comparison, the *λ′* of ICB was also determined and turned out to be 1.33:1, implying the incomplete inclusion that was consistent with its SEM image (Figure 1d).

### 3.3. Structure Characterization

#### 3.3.1. IR, XRD, and DSC Characterization

To study the particle structures, products of different morphologies (i.e., BP_n_, Sample 2; BP_mix_, Sample 4; BP_m_, Sample 7) were analyzed using IR, XRD, and DSC, as well as raw BBR and β-CD, a physical mixture (1:1, *n*/*n*), and ICB for comparison.

**Figure 4:**
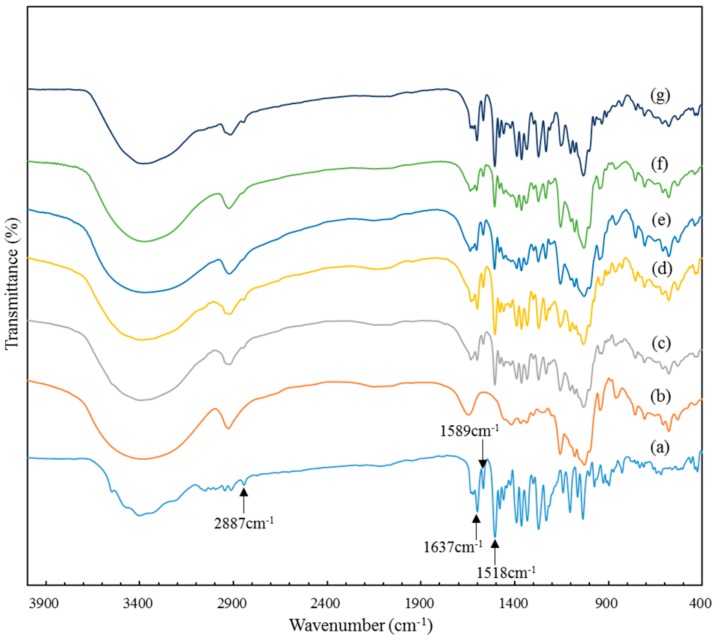
Infrared reflection spectra of (**a**) BBR; (**b**) raw β-cyclodextrin; (**c**) physical mixture (1:1, *n*/*n*); (**d**) ICB; (**e**) (BP_m_ Sample 7; (**f**) BP_n_ Sample 2#; (**g**) BP_mix_ Sample 4.

The IR spectrum of BBR (Figure 4a) indicated the existence of a methoxy group (2887 cm^−1^) and an iminium double bond (C=N^+^, 1637 cm^−1^), while the signals at 1589 and 1518 cm^−1^ represented the aromatic C=C bending and the furyl group, respectively [37]. β-CD exhibited a similar spectrum to that reported [37]. The typical vibration absorption band of BBR could clearly be detected in the physical-mixture spectrum (Figure 4c), as well as ICB (Figure 4d), which indicated the existence of free BBR. The mixed-morphology IR curve (Figure 4g) also revealed the existence of a mass of free BBR, in accordance with its SEM image (Figure 2d). However, in the spectra of BPs appearing as micron spheres (Figure 4e) or nanospheres (Figure 4f), the peak at 2887 cm^−1^, corresponding to the methoxy group stretching, disappeared, indicating a possible interaction of the guest molecule (BBR) in the host cavity (β-CD) to form the inclusion complex.

**Figure 5:**
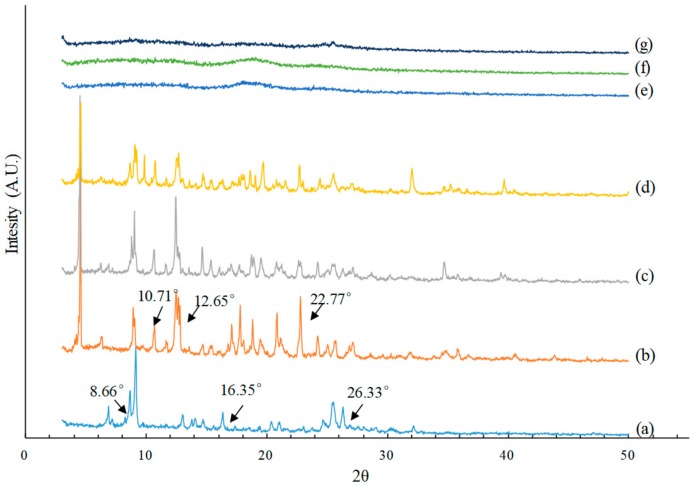
X-ray diffraction patterns of (**a**) BBR; (**b**) raw β-cyclodextrin; (**c**) physical mixture (1:1, *n*/*n*); (**d**) ICB; (**e**) BP_m_ Sample 7; (**f**) BP_n_ Sample 2; (**g**) BP_mix_ Sample 4.

The XRD patterns revealed the crystalline nature of BBR (Figure 5a) with high intensity peaks at 8.66°, 16.35°, and 26.33° [38], and β-CD (Figure 5b) at 10.71°, 12.65°, and 22.77°. The presence of both crystals in the physical mixture could be confirmed (Figure 5c) by the coexistence of characteristic peaks of BBR and β-CD. Nevertheless, the ICB pattern (Figure 5d) showed less intense peaks at 16.35° and 26.33°, indicating a reduction of BBR crystallinity. In contrast, all BP samples obtained via SEDS (Figure 5e–g) exhibited no characteristic diffraction peaks; manifested amorphous states were formed for the hybrid of BBR and β-CD after being processed via SEDS. The amorphous BP state might have been caused by the very high supersaturation degree formed during the SEDS process, which led to splitting nucleation speed and seriously restricted the crystal growth of BBR and β-CD.

**Figure 6:**
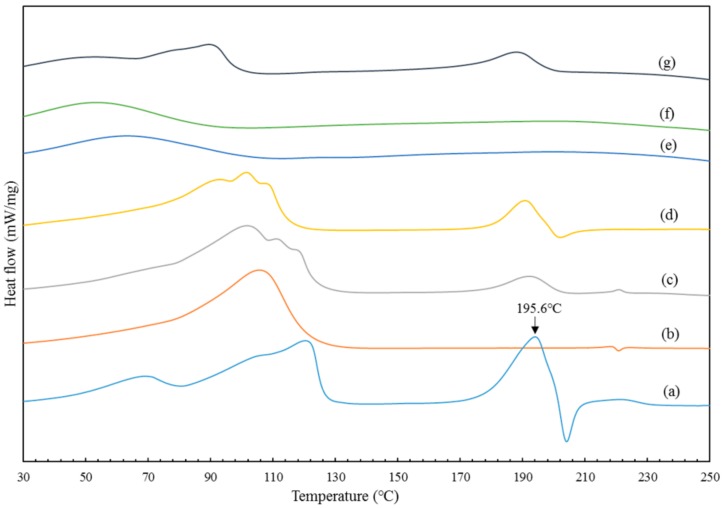
Differential thermal scanning curves of (**a**) BBR; (**b**) raw β-cyclodextrin; (**c**) physical mixture (1:1, *n*/*n*); (**d**) ICB; (**e**) (BP_m_ Sample 7; (**f**) BP_n_ Sample 2; (**g**) BP_mix_ Sample 4.

The DSC curves are given in Figure 6. The BBR thermogram (Figure 6a) showed a sharp endothermic peak at 195.6 °C, corresponding to the melting point of BBR in crystalline form. β-CD (Figure 6b) exhibited a very broad endothermic peak between 70 and 130 °C due to the loss of water molecules from the cyclodextrin cavity [39]. The fusion endothermic peak of BBR in the thermal curve of the physical mixture (Figure 6c) was much lower than that of the independent sample and shifted to a lower temperature because of the interaction between BBR and β-CD [40]. ICB (Figure 6d) was identical to the physical mixture, except the phase transformation temperature was lower, which indicated that crystallinity was transformed, but free BBR still existed. BP_mix_ (Figure 6g) was also demonstrated to have free crystalline BBR. However, the thermal curve of the other two types of BP (Figure 6e,f) showed complete disappearance of the BBR endothermic peaks, indicating the formation of an amorphous form.

#### 3.3.2. Intermolecular Interaction of BBR and β-CD in BP_n_

To research the possible inclusion mode of BP, the ^1^H NMR spectra of β-CD in the presence (in the form of BP_n_) or absence of BBR were investigated. Because the H-3, H-5, and H-6 protons of β-CD are located in the semipolar interior cavity, and the other protons (H-1, H-2, H-4) are in the hydrophilic exterior fringe of the β-CD cavity, the presence of BBR in β-CD could be proved through the chemical shifts of β-CD at H-3, H-5, and H-6 protons [41]. As Table 2 shows, H-3, H-5, and H-6 protons of β-CD had relatively higher chemical shifts compared to other protons in the ^1^H NMR spectra of both BP_n_ and ICB, confirming the formation of inclusion complexes between BBR and β-CD.

**Figure 7:**
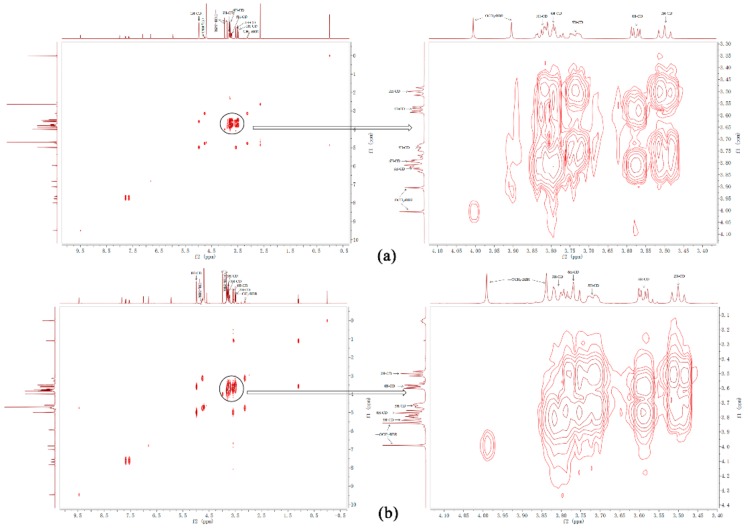
NOESY spectrum of (**a**) BP_n_ Sample 2, and (**b**) ICB.

Two-dimensional ^1^H NMR, a powerful tool for investigating inter- and intramolecular interactions, was used in this study. The presence of NOE cross-peaks between the protons from two different species indicated spatial contact within 0.4 nm and provided effective message to study the spatial conformations of inclusion complex [20]. 2D NOESY of BP_n_ and ICB in D_2_O was shown in Figure 7. Both in Figure 7a,b, cross-peaks were observed between the methoxy group of BBR and the H-3 and H-5 protons of β-CD, indicating that BBR was included into the hydrophobic cavity of β-CD through the methoxy group side. Slight cross-peaks between the methoxy group of BBR and the H-2 and H-4 protons of β-CD appeared in ICB (Figure 7b), manifesting part of the free BBR that existed and had weak acting force on the surface of the hydrophilic shell of β-CD.

### 3.4. Dissolution Enhancement and Morphological Dependence

**Figure 8:**
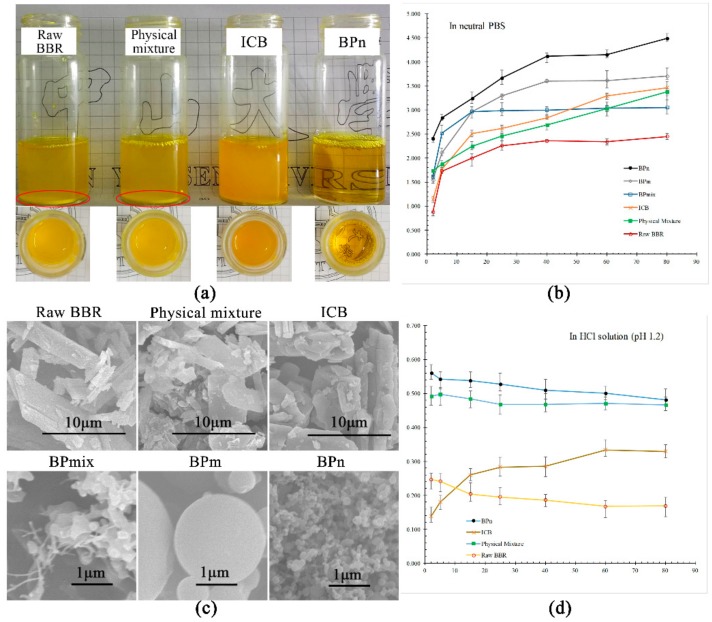
(**a**) BBR liquids, physical mixture of BBR and β-CD, ICB, and BPs added at an amount equivalent to 3.5 mg/mL BBR under room temperature; (**b**) dissolution curves of raw BBR, physical mixture, ICB, BP_mix_ (Sample 4), BP_m_ (Sample 7), and BP_n_ (Sample 11) in neutral PBS; and (**c**) their SEM images; (**d**) dissolution curves in acidic aqueous (HCl, pH 1.2) of raw BBR, physical mixture, ICB, and BP_n_ (Sample 11).

Dissolution studies of BBR for the BPs of the three morphologies (BP_mix_ #4, BP_m_ #7, and BP_n_ #11) were separately investigated in neutral phosphate-buffered saline (PBS) and an HCl solution (pH 1.2). Again, the raw materials, including their physical mixture and ICB, were required for contrast. The dissolution curves are shown in Figure 8.

As shown in Figure 8a, at a certain addition amount (equivalent to 3.5 mg/mL BBR) of raw BBR, the physical mixture of BBR and β-CD, ICB, and BP_n_ (#11) in water under room temperature, turbid liquids were produced for the former three, whereas a transparent solution could be obtained for BP_n_, demonstrating a higher solubility of BBR in BP_n_. The homogeneous suspension of ICB, different from the precipitation-existing liquids of BBR and the physical mixture, also implied that undissolved ICB had better dispersive stability than free BBR in water. Curves in Figure 8b exhibit the comparison of BBR dissolution properties in PBS between raw BBR, physical mixture, ICB, and BPs with different morphologies. The solubilities of BBR at 80 min increased in sequence as follows: raw BBR < BP_mix_ (#4) < physical mixture < ICB < BP_m_ (#7) < BP_n_ (#11). The solubilities of BP_n_ at 2 and 80 min were improved to 2.40 ± 0.01 and 4.49 ± 0.03 mg/mL, from 0.87 ± 0.02 and 2.45 ± 0.02 mg/mL of raw BBR, respectively, indicating significant improvements of both the dissolution rate and saturated solubility of BBR. 

Considering the SEM images in Figure 8c, it was obvious that the dissolution properties of BBR greatly depended on particle morphology and structure. Based on the results above, two conclusions could be drawn: (i) The structure of inclusion complexes improved the dissolution rate and solubility of BBR, but only to some extent. It has also been reported that BBR solubility for ICB using β-CD reached its saturation value at about 3.46 mg/mL [42]; (ii) The dissolution property could further be improved by reducing particle size (comparing ICB, BP_m_, and BP_n_). Inclusion structure especially helped BBR disperse in molecular form, while the decrease of particle size allowed an increased surface area for the drug to be more available for solvation [43,44]. The increased surface area was also in favor of the release of BBR, leading to a faster dissolution rate.

An interesting result was that the dissolution curve of the physical mixture approximated that of ICB, with only slightly less solubility. This may be because inclusion structures were also formed via self-assembly during the water-bath stirring process for the physical mixture in dissolution determination [44]. Another noteworthy phenomenon was BP_mix_ #4 showing less final solubility than ICB despite smaller particle size, attributed to the lacking amount of β-CD (only about 1/5 of the amount in ICB), and free BBR crystals formed. However, its initial rapid release caused by the part of BBR included in β-CD proved the remarkable ability of BP to enhance dissolution.

In Figure 8d, the dissolution curves of raw BBR, physical mixture, ICB, and BP_n_ #11 in an HCl solution of pH 1.2 are displayed. Although the sequence of final solubilities (i.e., raw BBR < ICB < physical mixture < BP_n_) was like that in neutral PBS, all saturation solubility values sharply decreased, from 2.45 ± 0.02, 3.46 ± 0.01, 3.37 ± 0.15, and 4.49 ± 0.03 mg/mL in the neutral aqueous solution to 0.17 ± 0.01, 0.33 ± 0.02, 0.47 ± 0.01, and 0.48 ± 0.01 mg/mL. This was because BBR easily self-aggregated under acidic conditions due to its existence in an ionized form, leading to the decrease of BBR solubility [13]. Interestingly, the solubilities of all samples except ICB showed maximum values within the first 2 min and a decreasing trend with time. This phenomenon can be explained by a competition for BBR between the processes of dissolving and self-aggregation. For samples of raw BBR, physical mixture, and BP_n_, self-aggregation speeds were higher than their drug-release rate after the initial burst, decreasing overall solubility. In contrast, ICB had a slower dissolution rate at the beginning (also indicated by curves in PBS), keeping BBR concentration down in the solution, which was averse to self-aggregation and enabled a generally slight increase of BBR solubility over time. 

### 3.5. Pharmacokinetic Studies in Rats

**Figure 9:**
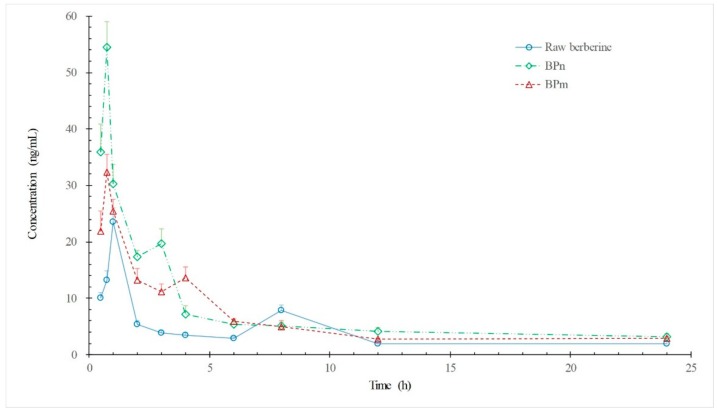
Plasma concentration profiles in rats after oral administrations of various formulations at a dose of 150 mg/kg. Values represent mean ± SD (*n* = 5). ◯, raw berberine; △, BP_m_; ⋄ BP_n_.

The comparative pharmacokinetic profiles of raw BBR, BP_m_, and BP_n_ after their oral administration are shown in Figure 9, and pharmacokinetic parameters are summarized in Table 3. All curves in Figure 9 exhibited double peaks, which may be due to the enterohepatic circulation of berberine. At 24 h after oral administration of raw berberine or BP, berberine plasma concentration was hardly detectable (below 2 ng/mL). *C*_max_ value increased in the sequence of raw BBR < BP_m_ < BP_n_, as well as at the value of *AUC*_0–t_. Furthermore, the *C*_max_ and *AUC*_0–t_ of BP_n_ were 2.32 and 1.86 times greater than that of raw BBR (Table 3). Meanwhile, compared to raw BBR, both the values of *T*_max_ and *t*_1/2_ were advanced for BP, and mean residence time (MRT) was shortened in step.

Results of pharmacokinetics studies indicate that, with enhanced dissolution properties, the oral bioavailability of BBR was improved and the entry of BBR into the bloodstream was accelerated. A dramatic finding was that values of relative bioavailability *F*_rel_ of BP_m_ (153.59 ± 11.53%) and BP_n_ (185.57 ± 13.49%) were highly consistent with the multiples of solubility increase versus the raw BBR of BP_m_ (1.51 times) and BP_n_ (1.83 times), respectively. Considering the complexity of drug adsorption, this numerical approximation may be a coincidence, but the positive effect of dissolution enhancement on BBR oral bioavailability can be concluded. However, the BBR bioavailability increase brought by BP was small. This may be because the solubility of BBR in water (about 2 mg/mL) does not make it a ‘poorly water-soluble drug’, and further dissolution enhancement of BBR is limited. Therefore, the major limiting factors for the very low oral bioavailability of BBR should be sought from its rapid metabolism or efflux. 

## 4. Conclusions

BP were prepared using SEDS, and the dissolution property of BBR was controlled by manipulating the particle morphology. Characterizations using XRD, IR, DSC, and 1H NMR demonstrated that an amorphous and inclusion structure was formed in BP_n_. Pharmacokinetics studies in rats showed that the oral bioavailability of BBR was increased in the sequence of raw BBR < BP_m_ < BP_n_, in keeping with the increase tendency of their dissolution properties. The entry of BBR into the BP bloodstream was also faster than that of raw BBR. The results indicate that dissolution enhancement was positive for the increase of BBR oral bioavailability, but the effect was limited. To further largely improve the BBR bioavailability, obstacles relating to drug efflux and metabolism should be overcome.

## Figures and Tables

**Table 1:** Operating parameters and BBR contents of a BP prepared via SEDS.

Sample No.	*λ* (*n*/*n*)	Temperature (°C)	Pressure (bar)	BBR Concentration (mg/mL)	*λ′* (*n*/*n*)	SEM Images
1	1:2	40	120	15	0.46	Figure 2a
2	1:1	40	120	15	1.02	Figure 2b
3	3:1	40	120	15	2.10	Figure 2c
4	5:1	40	120	15	3.52	Figure 2d
5	BBR only	40	120	15	\	Figure 2e
6	1:1	50	120	15	1.03	Figure 3a
7	1:1	60	120	15	0.93	Figure 3b
8	1:1	40	90	15	0.94	Figure 3c
9	1:1	40	150	15	1.03	Figure 3d
10	1:1	40	120	9	0.95	Figure 3e
11	1:1	40	120	3	0.88	Figure 3f

*λ*: initial mol ratio of BBR to β-CD; *λ′*: actual mol ratio of BBR to β-CD in BP.

**Table 2:** ^1^H NMR (600 MHz) chemical shift (δ) data (in ppm) of pure β-CD, BP_n_, and ICB.

		δ			Δδ	
		δ_(β-CD)_	δ_(BPn)_	δ_(ICB)_	δ_(BPn)_ − δ_(β-CD)_	δ_(ICB)_ − δ_(β-CD)_
H-1	d	3.99	4.00	3.99	0.01	0.00
H-2	t	3.51	3.50	3.50	−0.01	−0.01
H-3	t	3.89	3.83	3.82	−0.06	−0.07
H-4	dd	3.57	3.58	3.59	0.01	0.02
H-5	m	3.78	3.74	3.72	−0.04	−0.06
H-6	m	3.82	3.79	3.78	−0.03	−0.04

**Table 3:** Pharmacokinetic parameters of berberine for raw berberine, BP_m_, and BP_n_ in rat plasma.

Parameters	Raw Berberine	BP_m_	BP_n_
*C*_max_ (μg/mL)	25.5 ± 5.5	33.1 ± 5.9	54.4 ± 10.3
*T*_max_ (h)	1.0 ± 0.00	0.85 ± 0.14	0.75 ± 0.01
*AUC*_0→t_ (μg·L^−1^·h)	92.5 ± 9.6	142.1 ± 10.7	171.7 ± 12.5
*AUC*_0→__∞_ (μg·L^−1^·h)	149.7 ± 11.9	173.6 ± 5.0	207.1 ± 21.3
*K* (h^−1^)	0.06 ± 0.01	0.09 ± 0.02	0.09 ± 0.01
*t*_1/2_ (h)	12.70 ± 2.00	7.54 ± 1.42	7.88 ± 1.50
*CL* (L·kg^−1^·h^−1^)	67.15 ± 5.42	57.63 ± 1.63	48.68 ± 4.87
*MRT* (h)	18.33 ± 2.88	10.89 ± 2.05	11.38 ± 2.16
*F*_rel_ (%)	100	153.59 ± 11.53	185.57 ± 13.49
(*n* = 5, mean ± SD)			

## Explanation of Abbreviations and Symbols

**Abbreviation or Symbol**	**Full Name**
AUC	The area under the concentration–time curve
*AUC*_0→∞_	*AUC* from time zero to infinity
*AUC*_0→__t_	*AUC* from time zero to test time
BBR	berberine
BP	Berberine-loaded solid polymeric particle
BP_m_	BP of microsphere
BP_mix_	BP of mixed morphology
BP_n_	BP of nanosphere
*C*_max_	Peak concentration
DCM	Dichloromethane
DMSO	Dimethyl sulfoxide
*F*_rel_	Relative bioavailability
ICB	Traditional inclusion complexes of BBR
MRT	Mean residence time
PBS	Phosphate-buffered saline
SAS	Supercritical antisolvent process
SEDS	Solution-enhanced dispersion by supercritical fluids
*T*_max_	Time of peak concentration
β-CD	β-cyclodextrin
*λ*	Initial mol ratio of BBR to β-CD
*λ′*	Mol ratio of BBR to β-CD in product

## References

[1] Imenshahidi M., Hosseinzadeh H. (2016). Berberis Vulgaris and Berberine: An Update Review. Phytother. Res..

[2] Chao J., Dai Y., Verpoorte R., Lam W., Cheng Y.C., Pao L.H., Zhang W., Chen S. (2017). Major Achievements of Evidence-Based Traditional Chinese Medicine in Treating Major Diseases. Biochem. Pharmacol..

[3] Da Silva A.R., De Andrade Neto J.B., Da Silva C.R., Campos R.D.S., Costa Silva R.A., Freitas D.D., Do Nascimento F.B.S.A., De Andrade L.N.D., Sampaio L.S., Grangeiro T.B. (2016). Berberine Antifungal Activity in Fluconazole-Resistant Pathogenic Yeasts: Action Mechanism Evaluated by Flow Cytometry and Biofilm Growth Inhibition in Candida spp. Antimicrob. Agents Chemother..

[4] Wang Z., Chen Z., Yang S., Wang Y., Huang Z., Gao J., Tu S., Rao Z. (2014). Berberine Ameliorates Collagen-Induced Arthritis in Rats Associated with Anti-Inflammatory and Anti-Angiogenic Effects. Inflammation.

[5] Kuo C.L., Chi C.W., Liu T.Y. (2004). The Anti-Inflammatory Potential of Berberine in Vitro and in Vivo. Cancer Lett..

[6] Simoes-Pires C., Hostettmann K., Haouala A., Cuendet M., Falquet J., Graz B., Christen P. (2014). Reverse Pharmacology for Developing an Anti-Malarial Phytomedicine. The Example of Argemone Mexicana. Int. J. Parasitol. Drugs Drug Resist..

[7] Xiong X., Yang X., Liu Y., Zhang Y., Wang P., Wang J. (2013). Chinese Herbal Formulas for Treating Hypertension in Traditional Chinese Medicine: Perspective of Modern Science. Hypertens. Res..

[8] Ni W.J., Ding H.H., Tang L.Q. (2015). Berberine as a Promising Anti-Diabetic Nephropathy Drug: An Analysis of its Effects and Mechanisms. Eur. J. Pharmacol..

[9] Singh T., Vaid M., Katiyar N., Sharma S., Katiyar S.K. (2011). Berberine, an Isoquinoline Alkaloid, Inhibits Melanoma Cancer Cell Migration by Reducing the Expressions of Cyclooxygenase-2, Prostaglandin E2 and Prostaglandin E2 Receptors. Carcinogenesis.

[10] Ho Y.T., Yang J.S., Li T.C., Lin J.J., Lin J.G., Lai K.C., Ma C.Y., Wood W.G., Chung J.G. (2009). Berberine Suppresses in Vitro Migration and Invasion of Human SCC-4 Tongue Squamous Cancer Cells through the Inhibitions of FAK, IKK, NF-κB, u-PA and MMP-2 and -9. Cancer Lett..

[11] Kong W., Wei J., Abidi P., Lin M., Inaba S., Li C., Wang Y., Wang Z., Si S., Pan H. (2004). Berberine is a Novel Cholesterol-Lowering Drug Working through a Unique Mechanism Distinct from Statins. Nat. Med..

[12] Pirillo A., Catapano A.L. (2015). Berberine, a Plant Alkaloid with Lipid- and Glucose-Lowering Properties: From in Vitro Evidence to Clinical Studies. Atherosclerosis.

[13] Liu C.S., Zheng Y.R., Zhang Y.F., Long X.Y. (2016). Research Progress on Berberine with a Special Focus On its Oral Bioavailability. Fitoterapia.

[14] Guo S., Wang G., Wu T., Bai F., Xu J., Zhang X. (2017). Solid Dispersion of Berberine Hydrochlorid and Eudragit^®^ S100: Formulation, Physicochemical Characterization and Cytotoxicity Evaluation. J. Drug Deliv. Sci. Technol..

[15] Zhaojie M., Ming Z., Shengnan W., Xiaojia B., Hatch G.M., Jingkai G., Li C. (2014). Amorphous Solid Dispersion of Berberine with Absorption Enhancer Demonstrates a Remarkable Hypoglycemic Effect Via Improving its Bioavailability. Int. J. Pharmaceut..

[16] Fan D., Wu X., Dong W., Sun W., Li J., Tang X. (2013). Enhancement by Sodium Caprate and Sodium Deoxycholate of the Gastrointestinal Absorption of Berberine Chloride in Rats. Drug Dev. Ind. Pharm..

[17] Ma B.L., Yang Y., Dai Y., Li Q., Lin G., Ma Y.M. (2017). Polyethylene Glycol 400 (PEG400) Affects the Systemic Exposure of Oral Drugs Based on Multiple Mechanisms: Taking Berberine as an Example. RSC Adv..

[18] Chen W., Miao Y.Q., Fan D.J., Yang S.S., Lin X., Meng L.K., Tang X. (2011). Bioavailability Study of Berberine and the Enhancing Effects of TPGS On Intestinal Absorption in Rats. AAPS Pharmscitech.

[19] Zhang Y., Cui Y.L., Gao L.N., Jiang H.L. (2013). Effects of B-Cyclodextrin On the Intestinal Absorption of Berberine Hydrochloride, a P-glycoprotein Substrate. Int. J. Biol. Macromol..

[20] Shen Z., Qin Q., Liao X., Yang B. (2017). Host-Guest Inclusion System of Glycyrrhetic Acid with Polyamine-B-Cyclodextrin: Preparation, Characterization, and Anticancer Activity. J. Mol. Struct..

[21] Braithwaite M.C., Kumar P., Choonara Y.E., du Toit L.C., Tomar L.K., Tyagi C., Pillay V. (2017). A Novel Multi-Tiered Experimental Approach Unfolding the Mechanisms Behind Cyclodextrin-Vitamin Inclusion Complexes for Enhanced Vitamin Solubility and Stability. Int. J. Pharmaceut..

[22] Zhu J.X., Tang D., Feng L., Zheng Z.G., Wang R.S., Wu A.G., Duan T.T., He B., Zhu Q. (2013). Development of Self-Microemulsifying Drug Delivery System for Oral Bioavailability Enhancement of Berberine Hydrochloride. Drug Dev. Ind. Pharm..

[23] Wang T., Wang N., Song H., Xi X., Wang J., Hao A., Li T. (2011). Preparation of an Anhydrous Reverse Micelle Delivery System to Enhance Oral Bioavailability and Anti-Diabetic Efficacy of Berberine. Eur. J. Pharm. Sci..

[24] Xue M., Yang M.X., Zhang W., Li X.M., Gao D.H., Ou Z.M., Li Z.P., Liu S.H., Li X.J., Yang S.Y. (2013). Characterization, Pharmacokinetics, and Hypoglycemic Effect of Berberine Loaded Solid Lipid Nanoparticles. Int. J. Nanomed..

[25] Meng X.P., Wang X., Wang H., Chen T., Wang Y., Wang Z. (2016). In Vitro Antitumor Efficacy of Berberine: Solid Lipid Nanoparticles Against Human HepG2, Huh7 and EC9706 Cancer Cell Lines. Colloidal Nanoparticles for Biomedical Applications XI.

[26] Esfandiari N. (2015). Production of Micro and Nano Particles of Pharmaceutical by Supercritical Carbon Dioxide. J. Supercrit. Fluids.

[27] Campardelli R., Baldino L., Reverchon E. (2015). Supercritical Fluids Applications in Nanomedicine. J. Supercrit. Fluids.

[28] Jia J., Wang J., Zhang K., Zhou D., Ge F., Zhao Y. (2017). Aescin Nanoparticles Prepared Using SEDS: Composition Stability and Dissolution Enhancement. J. Supercrit. Fluids.

[29] Girotra P., Singh S.K., Nagpal K. (2013). Supercritical Fluid Technology: A Promising Approach in Pharmaceutical Research. Pharm. Dev. Technol..

[30] De Marco I., Prosapio V., Cice F., Reverchon E. (2013). Use of Solvent Mixtures in Supercritical Antisolvent Process to Modify Precipitates Morphology: Cellulose Acetate Microparticles. J. Supercrit. Fluids.

[31] Rossmann M., Braeuer A., Leipertz A., Schluecker E. (2013). Manipulating the Size, the Morphology and the Polymorphism of Acetaminophen Using Supercritical Antisolvent (SAS) Precipitation. J. Supercrit. Fluids.

[32] Jia J., Song N., Gai Y., Zhang L., Zhao Y. (2016). Release-Controlled Curcumin Proliposome Produced by Ultrasound-Assisted Supercritical Antisolvent Method. J. Supercrit. Fluids.

[33] Miguel F., Martín A., Mattea F., Cocero M.J. (2008). Precipitation of Lutein and Co-Precipitation of Lutein and Poly-Lactic Acid with the Supercritical Anti-Solvent Process. Chem. Eng. Process. Process Intensif..

[34] De Marco I., Rossmann M., Prosapio V., Reverchon E., Braeuer A. (2015). Control of Particle Size, at Micrometric and Nanometric Range, Using Supercritical Antisolvent Precipitation from Solvent Mixtures: Application to PVP. Chem. Eng. J..

[35] De Marco I., Reverchon E. (2011). Influence of Pressure, Temperature and Concentration on the Mechanisms of Particle Precipitation in Supercritical Antisolvent Micronization. J. Supercrit. Fluids.

[36] De Marco I., Knauer O., Cice F., Braeuer A., Reverchon E. (2012). Interactions of Phase Equilibria, Jet Fluid Dynamics and Mass Transfer During Supercritical Antisolvent Micronization: The Influence of Solvents. Chem. Eng. J..

[37] Li N., Xu L. (2010). Thermal Analysis of β-cyclodextrin/Berberine Chloride Inclusion Compounds. Thermochim. Acta.

[38] Yu F., Li Y., Chen Q., He Y., Wang H., Yang L., Guo S., Meng Z., Cui J., Xue M. (2016). Monodisperse Microparticles Loaded with the Self-Assembled Berberine-Phospholipid Complex-Based Phytosomes for Improving Oral Bioavailability and Enhancing Hypoglycemic Efficiency. Eur. J. Pharm. Biopharm..

[39] Silva J.C., Soares J.M.D., Silva M.G.E., de Lavor É.M., Andrade V.M., Menezes P.D.P., Araújo A.A.D.S., Leite L.H.I., Menezes I.R.A.D. (2017). Docking, Characterization and Investigation of B-Cyclodextrin Complexed with Farnesol, an Acyclic Sesquiterpene Alcohol, Produces Orofacial Antinociceptive Profile in Experimental Protocols. Process Biochem..

[40] Battu S.K., Repka M.A., Maddineni S., Chittiboyina A.G., Avery M.A., Majumdar S. (2010). Physicochemical Characterization of Berberine Chloride: A Perspective in the Development of a Solution Dosage Form for Oral Delivery. AAPS Pharmscitech.

[41] Hazra S., Hossain M., Kumar G.S. (2014). Studies On A-, B-, and Γ-Cyclodextrin Inclusion Complexes of Isoquinoline Alkaloids Berberine, Palmatine and Coralyne. J. Incl. Phenom. Macrocycl. Chem..

[42] Ma S., Wang Y., Shang X., Yan F. (2012). Formulation of Berberine Hydrochloride and Hydroxypropyl-B-Cyclodextrin Inclusion Complex with Enhanced Dissolution and Reduced Bitterness. Trop. J. Pharm. Res..

[43] Bunjes H. (2010). Lipid Nanoparticles for the Delivery of Poorly Water-Soluble Drugs. J. Pharm. Pharmacol..

[44] Kalepu S., Nekkanti V. (2016). Improved Delivery of Poorly Soluble Compounds Using Nanoparticle Technology: A Review. Drug Deliv. Transl. Res..

